# Supplementary material for: The Regulation of MicroRNA-21 by Interleukin-6 and Its Role in the Development of Fibrosis in Endometriotic Lesions
Source: Int J Mol Sci. 2024 Aug 19;25(16):8994. doi: 10.3390/ijms25168994 (PMC11354763; doi:10.3390/ijms25168994)
Supplement: Supplementary file 1 [file ijms-25-08994-s001.zip › ijms-3099849-supplementary.pdf]

Supplementary Table S1. RT-qPCR primer list CUT&RUN.

Gene	Species	Primer Sequence
pSTAT3	Human	(F) 5′-TGCCTCCCAAGTTTGCTAATGC-3′<br>(R) 3′-ACAATCTGTGCGTCATCCTTATCC-5′

Supplementary Table S2. RT-qPCR primer list.

Gene	Species	Primer Sequences
CTGF	Human<br>Baboon	(F)5′-CAG CAT GGA CGT TCG TCT G-3′<br>(R)5′-AAC CAC GGT TTG GTC CTT GG-3′
CTGF	Mouse	F)5′-GGG CCT CTT CTG CGA TTT C-3′<br>(R)5′-ATC CAG GCA AGT GCA TTG GTA-3′
RPL17	Human<br>Baboon	(F)5′-ACG AAA AGC CAC GAA GTA TCT-3′<br>(R)5′GAC CTT GTC TCC AGC CCC AT-3′
18S	Human<br>Baboon	(F)5′-TGA TTA AGT CCC TGC CCT TTG T-3′<br>(R)5′-TCA AGT TGC ACC GTC TTC TCA G-3′
18S	Mouse	(F)5′- GTA ACC CGT TGA ACC CCA TT-3′<br>(R)5′CCA AAT CGG TAG TAG CG-3′
MiR-21	Human<br>Mouse<br>Baboon	UAGCUUAUCAGACUGAUGUUGA
U6	Human<br>Mouse<br>Baboon	GTGCTCGCTTCGGCAGCACATATACTAAAATT<br>GGAACGATACAGAGAAGATTAGCATGGCCCC<br>TGCGCAAGGATGACACGCAAATTCGTGAAGCGTTCCATATTT
SMAD7	Human	(F)5′-TTC CTC CGC TGA AAC AGG G-3′<br>(R)5′-CCT CCC AGT ATG CCA CCA C-3′
SMAD7	Mouse	(F)5′-GGC CGG ATC TCA GGC ATT C-3′<br>(R)5′-TTG GGT ATC TGG AGT AAG GAG G-3′
36B4	Mouse	(F)5′- CAT CAC CAC GAA AAT CTC CA-3′<br>(R)5′- TTG TCA AAC ACC TGC TGG AT-3′
